# Reconstruction method for massive lateral chest wall sarcoma using titanium plates and mesh: a case report

**DOI:** 10.1186/s13019-024-02639-5

**Published:** 2024-04-18

**Authors:** Shin Tanaka, Eiji Nakata, Toshifumi Ozaki, Shinichi Toyooka

**Affiliations:** 1 Department of General Thoracic Surgery and Breast and Endocrinological Surgery, Dentistry, and Pharmaceutical Sciences, Okayama University Graduate School of Medicine, Okayama, Japan; 2 Department of Orthopedic Surgery, Dentistry, and Pharmaceutical Sciences, Okayama University Graduate School of Medicine, 2-5-1, Shikata-cho, Kita-ku, Okayama, 700-8558 Japan

**Keywords:** Chest wall tumor surgery, Myxofibrosarcoma, Titanium plate, Gore-Tex patch, Case report

## Abstract

**Background:**

Very large chest wall resections can lead to acute thoracic insufficiency syndrome due to the interdependence of lung expansion and thoracic volume. Chest wall tumor surgeries often encounter complications, with the size of the chest wall defect being a significant predictor. Several methods for large chest wall reconstruction have been described, aiming to provide stability, prevent flail chest, and ensure airtight closure. However, no single method fulfills all requirements. Composite chest wall reconstruction using titanium plates and Gore-Tex patches has shown the potential to minimize physiologic abnormalities caused by extensive defects.

**Case presentation:**

A 42-year-old man with myxofibrosarcoma underwent multiple surgeries, chemotherapies, and radiation therapies due to repeated local recurrences. After right arm amputation and resection of the right third to fifth ribs, a local recurrence was detected. A 30 × 40 cm chest wall defect was resected en bloc, and a titanium plate was used for three-dimensional formability, preventing flail chest and volume loss. The Gore-Tex patch was then reconstructed into an arch shape, allowing lateral thoracic mobility. The patient recovered well and did not experience respiratory dysfunction or local recurrence but later succumbed to distant metastasis.

**Conclusions:**

In this case, the combination of a titanium plate and a Gore-Tex patch proved effective for reconstructing massive lateral chest wall defects. The approach provided stability, preserved thoracic volume, and allowed for lateral mobility. While the patient achieved a successful outcome in terms of local recurrence and respiratory function, distant metastasis remained a challenge for myxofibrosarcoma patients, and its impact on long-term prognosis requires further investigation. Nevertheless, the described procedure offers promise for managing extensive chest wall defects.

## Background

Very large chest wall resections can cause acute thoracic insufficiency syndrome because lung expansion and thoracic volume are interdependent. In chest wall tumor surgeries, the size of the chest wall defect is considered one of the most significant predictors of complications [1]. Various methods of large chest wall reconstruction have been described [2, 3]. The best method should provide stability of the chest, prevent flail chest, and ensure airtight closure. Marlex mesh, acrylic cement, polytetrafluoroethylene (Gore-Tex) patches, bone cement, fascia grafts, silicone implants, and titanium mesh alone or in various combinations have all been described for this purpose. All work reasonably well, although the choice of the appropriate reconstruction method depends on the location of the chest wall defect, and none of these solutions fulfill all the described requirements. Recently, a patient underwent a wide lateral chest wall resection for a myxofibrosarcoma with repeated local recurrence. In these cases, composite chest wall reconstruction using titanium plates and Gore-Tex patches could minimize the physiologic abnormalities caused by extensive chest wall defects.

## Case presentation

**Fig. 1:**
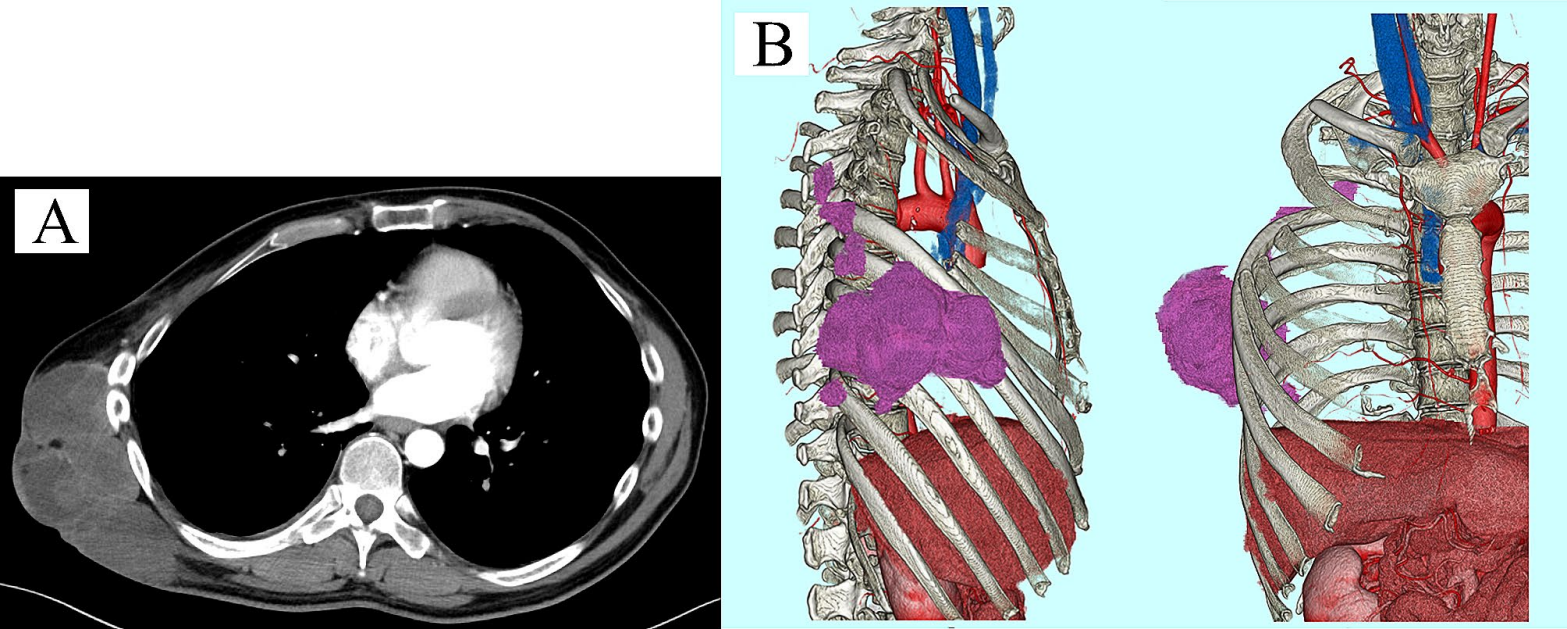
(**A**) Myxofibrosarcoma of the chest wall. (**B**) Local recurrence after right arm amputation and third to fifth rib resection

**Fig. 2:**
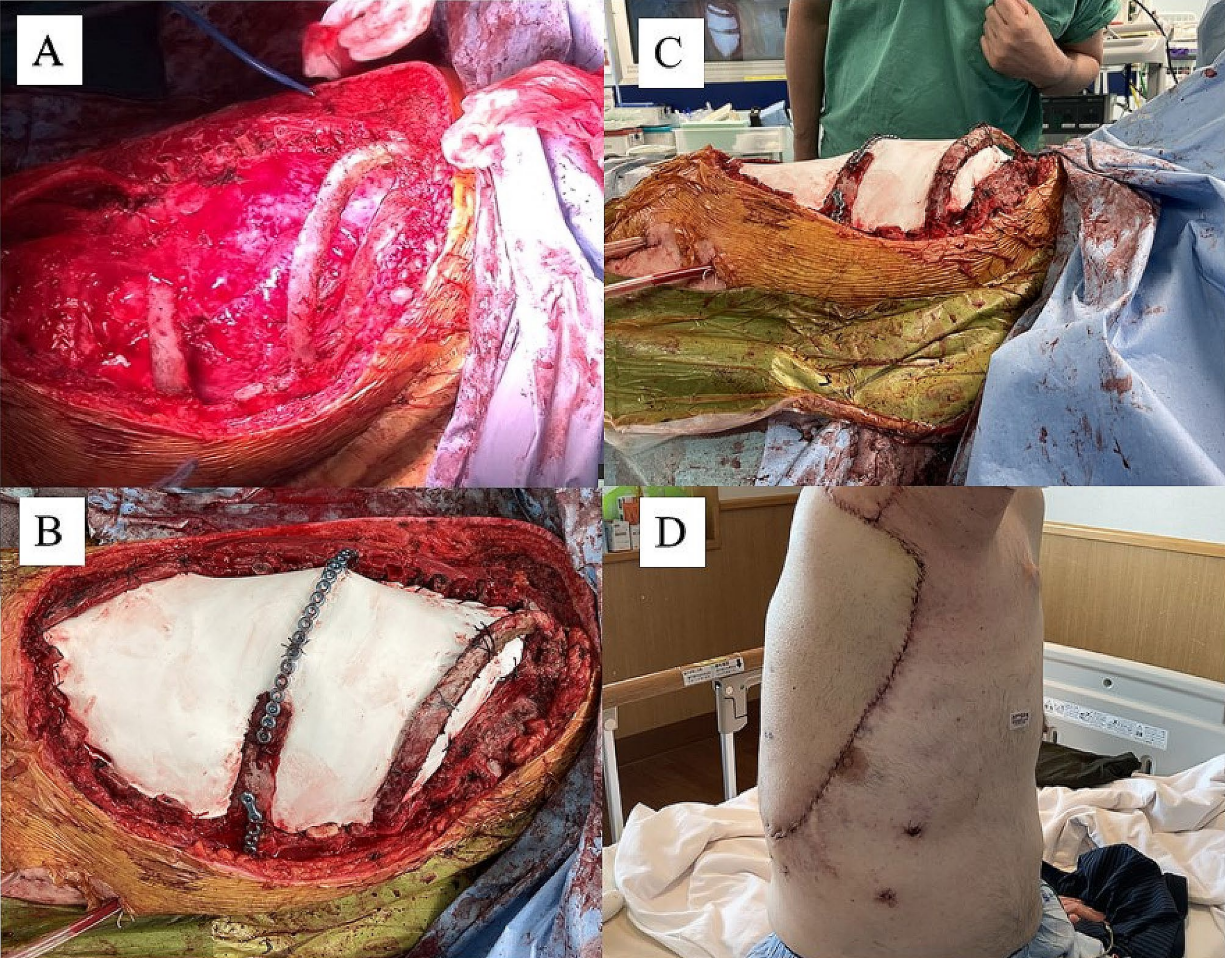
(**A**) En-bloc resection of the lateral chest wall. (**B**) Large lateral chest wall defect reconstruction. (**C**) Recreation of an arched thoracic cavity. (**D**) Closure of the skin defect using the anterolateral femoral skin flap

**Fig. 3:**
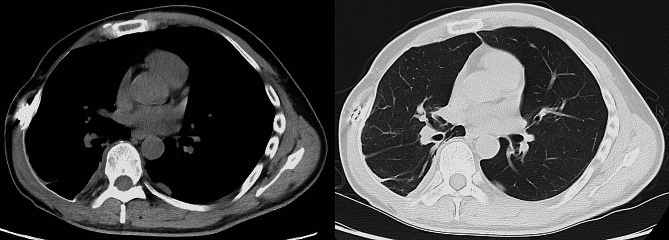
Postoperative CT scan. The combination of titanium plates and Gore-Tex patches creates a three-dimensional thorax

A 42-year-old man presented with a rapidly enlarging mass in his right axilla and was diagnosed with myxofibrosarcoma. In the 6 years leading up to this surgery, he had undergone a total of four surgeries, three chemotherapies which included doxorubicin hydrochloride, Ifosfamide, vincristine sulfate, cyclophosphamide and etoposide, and two radiation therapies due to repeated local recurrences. The patient ultimately underwent right arm amputation and a concomitant resection of the right third to fifth ribs. The chest wall was reconstructed using a Gore-Tex patch. Despite achieving R0 in the most recent surgery, local recurrence was discovered upon physical examination 3 months later. The computed tomography and the magnetic resonance imaging of the chest revealed a 21 × 19 × 6.7 cm right chest wall mass contiguous from previous resection margin (Fig. 1A, B, C), for which distant metastasis had been ruled out. En bloc tumor and chest wall resection was performed with the patient in the right lateral position under the single-lung ventilation. The latissimus dorsi muscle and almost the entire length of the third through ninth ribs except for the fourth rib, as well as those intercostal muscles with 3 cm margins from the tumor, were resected through the marginal skin incision. The chest wall now had a defect of almost the entire length of ribs 3–9, which was approximately 30 × 40 cm (Fig. 2A). We used a titanium plate because of the need for three-dimensional formability to prevent flail chest and intrapleural volume loss. The longest length of the plate was 18 cm; thus, it was impossible to reconstruct the entire length of the rib. Therefore, the plate was used only for the fourth rib; using this as a bridge, the Gore-Tex patch can be reconstructed into an arch shape. The defect was covered with the Gore-Tex patch, with tension fixed to the edges of the chest wall. The Gore-Tex patch was sewn to the plate and second rib after plate fixation to recreate an arched thoracic cavity (Fig. 2B, C). The anterolateral femoral skin flap using suprascapular artery and internal jugular vein was transposed to cover the skin defect (Fig. 2D). A chest tube was inserted into the thoracic cavity, and a drain was placed between the plate and flap. The patient was extubated the day after surgery. No flail chest was encountered postoperatively. To prevent infection related to the titanium plate, the drain on the plate was removed on the seventh postoperative day after the persistent fluid dried up [4]. Thirty days postoperatively, the patient did not require oxygen upon discharge (Fig. 3). After discharge, he returned to his normal daily life, although respiratory function tests could not be performedand, and he was doing well with no respiratory dysfunction and no local recurrence. No chemotherapy was administered after surgery. However, the patient died of distant metastasis 1 year postoperatively.

## Discussion and conclusions

In this case, the use of a titanium plate and Gore-Tex patch prevented chest wall instability, intrathoracic volume loss, and flail chest associated with a massive chest wall defect, while still allowing lateral thoracic mobility.

Myxofibrosarcoma is a distinctive subtype of soft tissue sarcoma characterized by a locally infiltrative behavior and a tendency for local recurrence. Margin-negative surgical resection is the mainstay of treatment for patients with soft tissue sarcoma. Local recurrence rates vary from 16 to 57%, and a significant proportion of patients experience multiple recurrences (25–52%). Radiotherapy is somewhat effective when employed as an adjuvant to resected margins. However, there is a lack of randomized data specifically assessing the role of radiation therapy in myxofibrosarcoma. Additionally, the role of chemotherapy in the treatment of myxofibrosarcoma remains less clear. Since the clinical course up to this point has indicated repeated local recurrence and no distant metastasis, the primary treatment was deemed to be surgery to resect the tumor with an adequate surgical margin.

The core principle of the reconstructive technique is to restore the maximum thoracic volume possible in a single step, thus avoiding the bidimensional thoracic deformity responsible for acute thoracic insufficiency syndrome. Furthermore, it should also allow for immediate extubation. Lardinois et al. [5] asserted that reconstruction of the chest wall plays a crucial role in determining postoperative morbidity and mortality. There are no established guidelines for reconstruction methods, and each surgical team chooses its own method depending on the location, size of the chest wall defect, and their own preference. In this case, the patient had a massive lateral wall defect with the entire length of the third to ninth ribs being absent. We selected a Gore-Tex patch for the base considering that the lateral thoracic region, unlike the anterior and dorsal regions, requires a certain degree of expansion and contraction due to motion; we selected a titanium plate because of the need for three-dimensional formability to prevent flail chest and intrapleural volume loss. Since the longest length of the plate is 18 cm, reconstructing the entire length of the rib was impossible. There have been reports of 3D printers being employed for thoracic reconstruction [6]. The optimal approach to ensure thoracic stability is to design and reconstruct all the resected ribs in each case. Unfortunately, this option was not available at our institution. Because of the short length of the existing plate, application of the plate to other ribs exhibiting full-length defects could compress the remaining lungs and subsequently reduce respiratory function.Therefore, the plate was used only for the fourth rib, which could be placed halfway through the reconstruction. Using this as a bridge, the second rib on the cephalic side and the tenth rib on the caudal side were used to reconstruct the Gore-Tex patch into an arch shape. The restoration of chest wall continuity and function relies on essential biomimetic reconstructions, but they frequently pose significant challenges due to the extensive nature of the defect. The new perspectives provided by three-dimensional formability may be a valid option for achieving good function and cosmetic results [6].

A clinical feature of myxofibrosarcoma is local recurrence because myxofibrosarcoma has an unusual infiltrative growth pattern along the fascial planes [7, 8]. In the present case, a large resection of the chest wall allowed local control of a tumor that had repeatedly recurred locally, but resulted in the death of the patient 1 year postoperatively due to distant lung metastases. Distant metastasis-free survival of myxofibrosarcoma patients was reported to be associated with mitotic activity and the margin status [9], and none of the risk factors for distant metastasis were met in the present case. Since repeated chemoradiotherapy has already been perfomed, we concluded that margin-negative surgical resection was feasible using the present reconstruction method. However, the early onset of distant metastases after surgery complicated the assessment of whether our chosen treatment strategy had a positive impact on the patient’s prognosis.

In conclusion, the titanium plate and Gore-Tex patch effectively reconstructed large chest wall defects, providing stability and lung volume preservation. Our patient achieved a successful outcome in terms of local recurrence and respiratory function, but distant metastasis remains a challenge, requiring further investigation. Nevertheless, the described procedure offers promise for managing extensive chest wall defects.

## Data Availability

Not applicable.
